# Chromogranin A and S100A12 protein measurement in hair of pigs: assay validation, changes across different production stages and comparison with other hair biomarkers

**DOI:** 10.1007/s11259-026-11404-z

**Published:** 2026-07-17

**Authors:** María Botía, Damián Escribano, Fernando Tecles, Silvia Martínez-Subiela, José Joaquín Cerón

**Affiliations:** 1 Salilab-UMU. Interdisciplinary Laboratory of Clinical Analysis (Interlab), Regional Campus of International Excellence ‘Campus Mare Nostrum’, University of Murcia, Murcia, 30100 Spain; 2 Department of Animal Production, Regional Campus of International Excellence ‘Campus Mare Nostrum’, University of Murcia, Campus de Espinardo S/N, Murcia, 30100 Spain

**Keywords:** Biomarkers, Hair, Non-invasive, Pig, Welfare

## Abstract

Hair has emerged as a non-invasive matrix for retrospective assessment of health status in pigs. While hair cortisol and sulphate dehydroepiandrosterone (DHEA-S) have been used as biomarkers of the hypothalamic-pituitary-adrenal (HPA) axis, indicators of the sympathetic-adrenomedullary (SAM) axis and innate immunity, such as chromogranin A (CgA) and S100A12, have not yet been measured in hair. This study aimed to (1) evaluate if CgA and S100A12 could be detected in pig hair, and (2) analyse possible longitudinal changes in their concentration across key production stages of growing pigs (lactation, nursery, and fattening in 20 pigs sampled longitudinally) and compare their values with cortisol and DHEA-S. For CgA and S100A12 measurements, AlphaLisa technology was used, while cortisol and DHEA-S were quantified using an automated chemiluminescence assay. Validation showed the immunoreactive detectability of CgA and S100A12 in hair extracts, with a high precision (CVs < 15%), linearity after serial sample dilution (R^2^ = 0.99), and adequate recovery (94.1–109.1%). During a productive cycle in fattening pigs, CgA concentrations increased progressively throughout the cycle, having higher values at the end of the fattening period (p=0.022), while S100A12 concentrations peaked significantly at the end of nursery (*p* = 0.0004). . Cortisol was highest at end of nursery, whereas DHEA-S increased significantly at the end offattening (*p* = 0.0002). This report provides evidence for the immunoreactive detectability of CgA and S100A12 in pig hair extracts, opening a new window into the potential evaluation of these analytes in this matrix.

## Introduction

Chromogranin A (CgA) is a glycoprotein secreted by neuroendocrine cells in response to activation of the sympathetic-adrenomedullary (SAM) axis (Escribano et al. 2019). Its quantification has been proposed as an useful biomarker of physiological and environmental challenges in the saliva of pigs (Huang et al. 2017) and humans (Tammayan et al. 2021). On the other hand, the protein S100A12, which belongs to the family of calcium-binding S100 proteins, has been linked to inflammatory processes and the activation of the innate immune system (Xia et al. 2024). In pigs, it has been observed that their concentrations may increase in saliva in situations of acute stress, such as during the transport of animals to the slaughterhouse (Botía et al. 2023).

In recent years, hair has emerged as a valuable biological matrix for assessing the health and welfare status in pigs. Unlike other biological samples such as blood or saliva (Ortín-Bustillo et al. 2023; Ott et al. 2014), which primarily reflect the current physiological state and are susceptible to acute changes, including those caused by handling and sampling procedures, hair provides a retrospective and cumulative record of hormone and protein secretion over extended periods, typically from one to two months (Bacci et al. 2014; Wiechers et al. 2021). This makes hair particularly suitable for evaluating sustained processes associated with development, adaptation and welfare (Casal et al. 2017; Wiechers et al. 2021). However, to the authors’ knowledge, the concentrations of CgA and S100A12 have not previously been quantified in hair.

The possibility of the measurement of these analytes in pigs hair would expand the number of biomarkers that can be evaluated in this sample type. Currently, cortisol and, with less frequency, dehydroepiandrosterone (DHEA), are the most commonly measured analytes in hair (Bergamin et al. 2019). Hair cortisol has been used as an indicator of long-term hypothalamic-pituitary-adrenal (HPA) axis activity (Levallois et al. 2024; Venegas et al. 2025). For its part, DHEA is a steroid that is primarily synthesised in the zona reticularis of the adrenal gland. It is a precursor of sexual hormones, such as androgens and oestrogens, and is considered an antagonist of cortisol (Lin et al. 2025). Furthermore, DHEA is largely found in circulation in its sulphated form, dehydroepiandrosterone sulphate (DHEA-S), which serves as a stable reservoir with a longer half-life and less rapid fluctuation than free DHEA. This conjugated form can be converted back to its active state in peripheral tissues, thereby contributing to the regulation of glucocorticoid activity and exerting anti-glucocorticoid, neuroprotective, and immunomodulatory effects (Labrie et al. 2001; Maninger et al. 2009).

Despite these advantages, the use of hair as a biomarker matrix presents some limitations. Although hair analysis is well-established for small lipophilic steroid hormones such as cortisol (Stalder and Kirschbaum 2012), the evaluation of proteins represents a significant challenge, since they can be tightly bound within the highly keratinized hair structure, making their efficient extraction without compromising their structural integrity a major methodological hurdle (Lee et al. 2006). Furthermore, the specific mechanisms driving the deposition of proteins and steroids into the hair shaft are not fully understood. While passive diffusion from the bloodstream into the growing follicle is widely considered the primary route, other factors such as local follicular synthesis or external contamination from sebum and environment cannot be ruled out (Heimbürge et al. 2020; Russell et al. 2012). Consequently, in the case of proteins, caution should be taken with the existing knowledge in translating hair analytes concentrations directly into an absolute measure of welfare.

Pig production involves successive stages characterised by marked changes in nutrition, social environment, housing conditions and management practices (Pedersen 2018). These stages represent distinct physiological contexts requiring continuous adaptation of neuroendocrine, immune, and metabolic systems (Maes et al. 2020; Tao et al. 2016). Therefore, the pig fattening cycle constitutes an appropriate biological model for investigating long-term physiological dynamics that could be reflected in pig hair. While cortisol and DHEA-S evaluate the HPA axis, CgA reflects the activity of the SAM axis, and S100A12 the innate immune system. As these physiological pathways have different functions and regulatory mechanisms, their combined evaluation may provide complementary information about the stress and inflammation responses experienced by pigs throughout their production cycle.

Therefore, the objectives of this study were (1) to evaluate if CgA and S100A12 could be detected in pig hair; (2) to analyse the potential of these analytes to reflect physiological differences throughout various production stages (end of lactation, the end of nursery, and the end of fattening), and compare their values with established hair biomarkers such as cortisol and DHEA-S; and (3) to assess the relationship between hair and salivary CgA concentrations, as well as the associations among all the evaluated hair biomarkers.

## Materials and methods

### Animals

The study involved 20 Large White pigs (*Sus scrofa domesticus*) (11 females and 9 males), from five litters (four piglets selected per litter) with an average size of 10.0 ± 0.7 piglets per litter. The trial was conducted at the Veterinary Teaching Farm of the University of Murcia (Guadalupe, Murcia, Spain). At birth, piglets presented a mean body weight of 1.93 ± 0.59 kg, with females averaging 1.83 ± 0.49 kg and males 2.02 ± 0.66 kg. The herd was certified free from porcine reproductive and respiratory syndrome virus, and all piglets received standard vaccinations at weaning against *Mycoplasma hyopneumoniae* (Stellamune Mycoplasma, Pfizer Animal Health, Madrid, Spain) and Porcine circovirus type 2 (Porcilis^®^ PCV, MSD Animal Health, Boxmeer, The Netherlands). All monitored animals were considered apparently healthy by veterinary inspection throughout the study, with no history of significant clinical disease or specific medical treatments administered during the sampling points. Growth performance was within the standard parameters for the genetic line.

The experimental procedures were conducted between October 2021 and April 2022 at the University of Murcia’s teaching farm, and the animal management and housing conditions were reported by Ortín-Bustillo et al. (2022b). While salivary results from this cohort were reported in that study, the hair samples collected concurrently were stored at room temperature in a dark place, as previously recommended (Berger et al. 2024) and were specifically analysed in 2025 for the present manuscript in order to evaluate the following biomarkers: CgA, S100A12, cortisol, and DHEA-S.

### Salivary CgA concentrations

To evaluate the relationship between transient and cumulative biomarker fluctuations, salivary CgA data were retrieved from our previous report (Ortín-Bustillo et al. 2022b). Briefly, salivary CgA was measured using an in-house time-resolved immunofluorometric assay (TR-IFMA) based on a species-specific antibody (Escribano et al. 2013). It was not possible to assess the correlation of salivary S100A12 concentrations due to insufficient surplus saliva samples being available.

### Experimental design

Hair samples were collected at three key production stages:

(i) End of lactation (L; 4 weeks old): All animals were individually identified by an ear tag. From 10 days of age, suckling piglets had access to a commercial pre-starter diet. Weaning was performed at 28 days after birth.
(ii) End of the nursery (N; 11 weeks old): following weaning, the piglets were transferred to an environmentally controlled nursery in a different building in the same farm for seven weeks. Each pen contained a standard feeder and a nipple drinker, providing *ad libitum* access to feed and water. Piglets were fed using a two-phase feeding programme over a seven-week period. Pre-starter (for the first 2 weeks, containing 10.63 MJ/kg Net energy (NE) and 183.2 g/kg crude protein (CP)) and starter diet (for the next 5 weeks, containing 10.55 MJ/kg NE and 172.2 g/kg CP).
(iii) End of the fattening (F; 25 weeks old). Pigs were then moved to fattening pens for 14 weeks. During this period, animals were given ad libitum access to a nutritionally balanced diet and water. The pigs were fed an initial growing diet (first 8 weeks, 10.26 MJ/kg NE, 164 g/kg CP) and a finishing diet (for the next 7 weeks, 10.40 MJ/kg NE and 149.6 g/kg CP).

### Hair processing and extraction

The hair was cut using an electric shaver by shaving an area of around 10 cm × 10 cm located in the dorsal area of the neck. This procedure was performed in accordance with a previous report (Casal et al. 2017). To ensure a standardized retrospective window of biomarker accumulation across the varying durations of the production stages, 1 cm segments of hair were collected, which represent approximately the length of hair grown during the three weeks preceding the sampling day (Kalliokoski et al. 2019). Then, the samples were placed in small plastic bags, individually marked and stored in a dark, dry environment at room temperature .

Hair samples were processed following the foundational protocol described by Davenport et al. (2006), which has been previously validated and successfully applied in porcine models for washing (Bacci et al. 2014) and extraction (Trevisan et al. 2017) procedures. The hair samples obtained from the pigs were weighed (250 mg), transferred into a polypropylene tube, and then covered with isopropanol (5 mL) for washing prior to extraction. The tube was then subjected to a mixing process at room temperature (RT), followed by a centrifugation step at 1500 g for a duration of one minute. The isopropanol was subsequently removed. The samples were then washed again with isopropanol, after which they were left at room temperature until completely dry. The hair samples were then divided into small pieces (approximately 1–2 mm), with 60 milligrams from each sample, placed in tubes with balls and pulverised to a fine powder in a homogeniser (Precellys Evolution homogeniser, Bertin Technologies, France). The pulverised hair was then incubated in 1 mL of methanol for 18 h at room temperature. This was done with continuous, gentle agitation to facilitate the extraction of any protein present. The samples were then subjected to a centrifugal process (2000 g, 5 min). After that, 0.6 mL of each methanol extract was transferred to a fresh Eppendorf tube and wassubjected to a process of evaporation, leading to the attainment of a dry state. This was achieved using a Speed Vac Concentrator (Concentrator 5301, Eppendorf). The dry extracts were then reconstituted with 0.1 mL of phosphate-buffered saline (PBS) and stored at − 80 °C to ensure the structural stability of the proteins and steroids prior to analysis (Pavani et al. 2021).

The research protocols were approved by the Bioethical Commission of Murcia University according to the European Council Directives regarding the protection of animals used for experimental purposes (Approval number: 235/2016; Approval date: 25 April 2016).

### Assay for S100A12 measurement

S100A12 was measured using a two-step, direct sandwich assay that was developed in 96-well plates, with a total sample volume of 15 µL per well. The assay is based on a method that had previously been validated for porcine saliva samples using AlphaLisa technology (Ortín-Bustillo et al. 2023) with some modifications . As the commercially available pure S100A12 protein (Pig S100 Calcium Binding Protein A12 (S100A12); abx166045, Abbexa Ltd) was not recognized by the specific antibody used in our assay, a standard curve consisting of eight points (from 0 to 208.4 ng/mL), was generated from a pooled hair sample measured with a specific commercially available assay (SED080Po Cloud-Clone, Katy, TX, USA). This commercial assay was previously validated and provided an intra- and inter-assay imprecision lower than 15% and was linear after serial sample dilutions (López-Martínez et al. 2023). Samples were incubated for 30 min at RT with 15 µL of acceptor beads (AlphaScreen Unconjugated Acceptor Beads, PerkinElmer, Waltham, MA, USA) coated with a commercial polyclonal antibody against porcine S100A12 (abx129972, Abbexa Ltd., Cambridge, UK) (12 µg/mL) . The second incubation was performed with 10 µL of the same polyclonal antibody biotinylated with a 20-fold molar excess (EZ-Link™, Micro Sulfo-NHS-Biotin, No-Weight™ Format, Thermo Scientific, Waltham, MA, USA) added to each well and incubated at RT for 30 min. As a final step, 10 µL of streptavidin-coated donor beads (20 µg/mL) were added to each well and incubated for 30 min at RT in the dark. The EnSpire^®^ Alpha Multimode Plate Reader (PerkinElmer, Waltham, Massachusetts, USA) was used to measure fluorescence intensity. S100A12 concentrations were expressed as pg per mg of hair.

### Assay for CgA measurement

Chromogranin A was measured using a direct competitive assay developed in 96-well plates, with a total sample volume of 15 µL per well. The standard curve consisted of eight points and was generated using CgA conjugated to bovine serum albumin (BSA) (synthesized by GenScript, Piscataway, NJ, USA) (from 0 to 500 ng/mL). The samples were incubated for 30 min at RT with 15 µL of acceptor beads (AlphaScreen Unconjugated Acceptor Beads, PerkinElmer, Waltham, MA, USA) coated with a monoclonal antibody against CgA (12 µg/mL) . A second incubation was performed by adding 10 µL of biotinylated CgA-BSA to each well and incubated at RT for 30 min. Finally, 10 µL of streptavidin-coated donor beads (10 µg/mL) were added to each well and incubated for 30 min at RT in the dark. The EnSpire^®^ Alpha Multimode Plate Reader (PerkinElmer, Waltham, Massachusetts, USA) was used to measure fluorescence intensity. CgA concentrations were expressed as pg per mg of hair.

### Cortisol and DHEA-S measurement

Cortisol and DHEA-S were also evaluated for comparative purposes with an automated chemiluminescent immunoassay (Cortisol and DHS, Immulite 2000, Siemens Medical Solutions Diagnostics). Cortisol and DHEA-S concentrations were expressed as pg per mg of hair.

### Analytical validation of S100A12 and CgA in pig hair

For analytical validation of the assays, imprecision was calculated as inter- and intra-assay variations and expressed as coefficients of variation (CVs). Five replicates of two samples at high and low analyte concentrations were analysed within the same run to determine the intra-assay precision or repeatability of the method. Five aliquots of each sample were stored in plastic vials at − 80 °C. These aliquots were measured in duplicate five times over five different days using freshly prepared calibration curves for inter-assay precision. The intra- and inter-assay CVs were calculated by dividing the standard deviation (SD) by the mean of the values of the averaged duplicate measurements each day and expressed as a percentage (CV% =SD/mean*100).

The accuracy and potential matrix effects within the hair extracts were assessed indirectly via linearity under dilution and a recovery test. For this, two samples at high and low concentration of each analyte were serially diluted from 1:4 to 1:32 with the assay buffer. In addition, a recovery test was also made after the hair extraction process in endogenous hair samples. Briefly, different concentrations of CgA-BSA and a serum sample with a known S100A12 concentration obtained by serial dilution (starting from 500 ng/mL and 31.7 ng/mL, respectively) were added independently to separate aliquots of the extracted hair samples to evaluate each analyte individually. To maintain a uniform matrix background, the extracted hair samples were kept at a constant dilution (1:4), and different amounts of spike were added. The percent recovery was calculated using the following formula: Measured concentration of spiked sample/Expected concentration of spiked sample*100. The acceptance criteria for recovery were set between 80% and 120%.

The limit of detection (LOD) and the lower limit of quantification (LLQ) were determined in order to evaluate the sensitivity of the method. The LOD was calculated as the mean of 10 replicate measurements of the blank plus three standard deviations. For the LLQ, a serial dilution (from 1:2 to 1:256) of a low-concentration extracted hair sample was performed and analysed in five replicates within the same run. The CV was calculated for each dilution to establish the LLQ as the lowest dilution that could be measured with less than 20% imprecision.

All analytical validation data associated tables are reported as assay-well concentrations (ng/mL) to reflect the method’s technical limits, prior to their mathematical conversion into hair concentrations (pg/mg) for the presentation of the results.

### Data analysis

Routine descriptive statistical procedures and computer software (Microsoft Excel 2025, Microsoft 365) were used to calculate means, medians, intra- and inter-assay CVs, and linearity. Statistical analysis of the biomarkers results was performed using GraphPad Prism 9 (GraphPad Software Inc., San Diego, USA). The sample size (*n* = 20; 60 total observations) was justified by a post-hoc power analysis, which for a repeated-measures design with three time points and a large effect size (f = 0.40) gave a statistical power of 0.84.

**Fig. 1:**
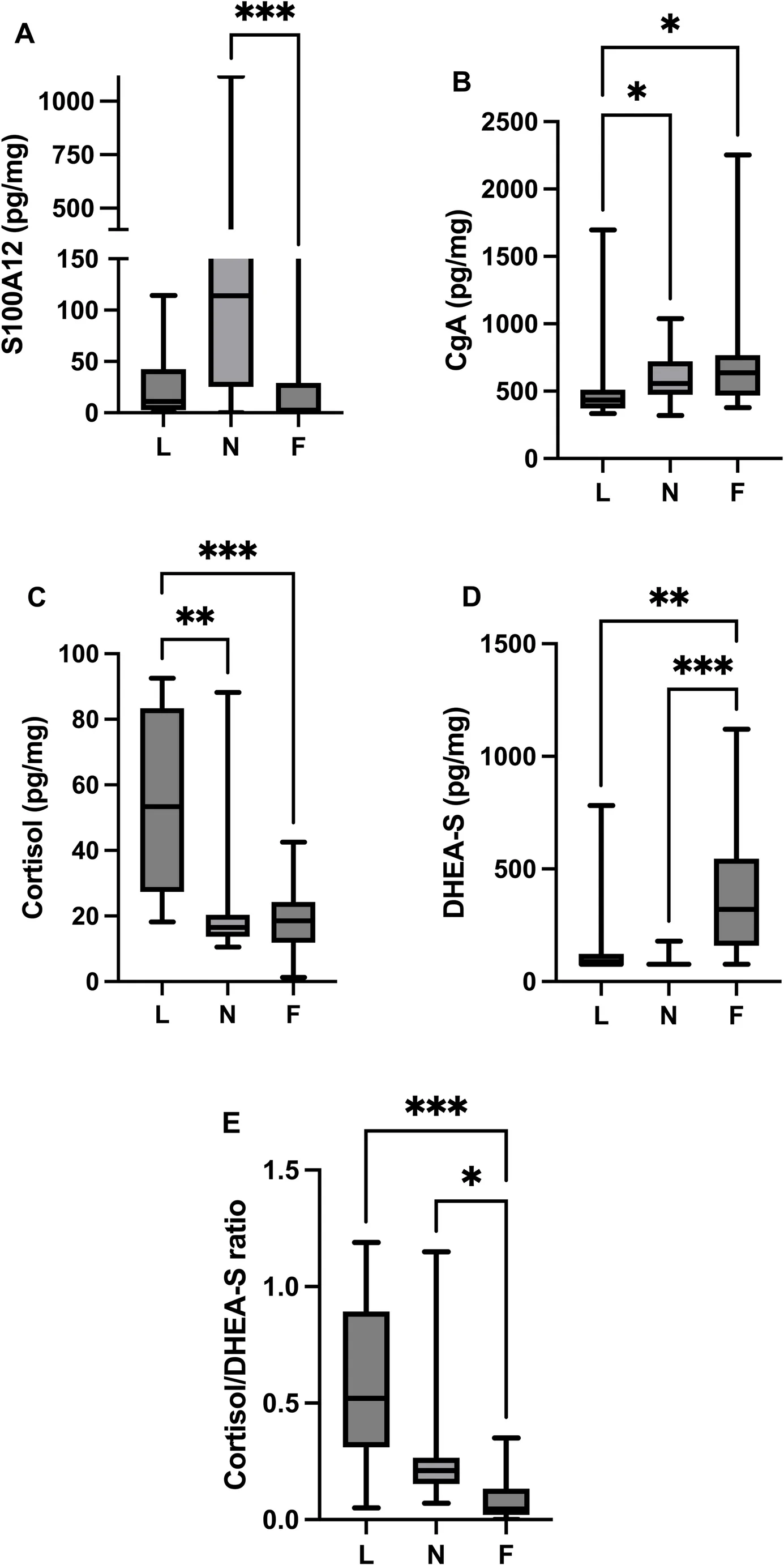
Changes in S100A12 (**A**), CgA (**B**), cortisol (**C**), DHEA-S (**D**), and cortisol/DHEA ratio (**E**) in hair samples obtained at the end of lactation (L), nursery (N) and fattening (**F**). Asterisks indicate significant differences (*p < 0.05; ** *p* < 0.01; *** *p* < 0.001)

Data distribution was assessed using the Shapiro–Wilk test. As not all biomarkers met the assumptions of normality, differences between production stages were analysed using the Friedman test for repeated measures, followed by Dunn’s post-hoc test for multiple comparisons. In addition, to evaluate the influence of sex and its potential interaction with production stage, data were further analysed using a mixed-effects model (restricted maximum likelihood, REML), with production stage included as a repeated-measures factor, sex as a fixed effect, and animal identity as a random effect. This approach allows the analysis of repeated-measures data with unbalanced group sizes and missing values. Results are presented as median and 25-75th percentile in Fig. 1. Median, 95% confidence intervals (CIs), and 25-75th percentiles are provided in Table 5 to describe the magnitude of the changes. Statistical significance was set at *p* < 0.05. The Spearman test (r) for non-parametric data was used to assess correlations between salivary and hair CgA, as well as between hair biomarkers. Finally, to avoid data loss, samples with concentrations below the lower limit of quantification (LLOQ) were assigned the LLOQ value for statistical analysis. This occurred in 5 of 20 samples measured for DHEA-S, particularly at end of nusery.

## Results

### Analytical validation of S100A12 and CgA in pig hair

In the analytical validation, these methods showed intra- and inter-assay CVs < 15%, as shown in Table 1.

**Table 1:** Intra- and inter-assays coefficients of variation (CV) for S100A12 and CgA quantification in porcine hair extracts

Analyte	Sample	Intra-Assay			Inter-Assay		
		Mean (ng/ml)	SD*	CV (%)	Mean (ng/ml)	SD*	CV (%)
S100A12	Low concentration	1.06	0.09	8.49	1.23	0.08	9.84
	High concentration	21.1	1.95	4.74	22.8	0.87	3.81
CgA	Low concentration	8.91	0.56	6.28	8.91	1.11	12.4
	High concentration	76.6	2.11	2.75	76.6	3.26	4.25

*SD: standard deviation.

Serial dilution of extracted hair samples demonstrated linearity (R^2^ = 0.99) for both analytes. The specific linear regression equations obtained were y = 1.0019x – 0.1227 and y = 1.0398x – 0.0547 for high and low S100A12 concentrations, respectively, and y = 0.9758x + 1.3304 and y = 0.9683x + 1.0375 for high and low CgA concentrations, respectively. The detailed expected, measured, and back-calculated concentrations, alongside the dilutional recoveries for each step, are presented in Table 2.

**Table 2:** Linearity under dilution for CgA and S100A12 in porcine hair extracts

Analyte	Sample	Dilution Factor	Expected Concentration (ng/mL)	Measured Concentration (ng/mL)	Back-calculated Concentration (ng/mL)	Recovery (%)
S100A12	High concentration	1:4	23.6	23.6	94.4	-
		1:8	11.8	12.2	97.6	103.4
		1:16	5.91	5.85	93.6	98.9
		1:32	2.96	3.09	98.8	104.4
	Low concentration	1:4	1.27	1.27	5.08	-
		1:8	0.64	0.67	5.36	104.7
		1:16	0.32	0.36	5.76	112.5
		1:32	0.16	0.20	6.40	125
CgA	High concentration	1:4	164.4	164.36	657.6	-
		1:8	82.2	73.6	588.8	89.5
		1:16	41.1	47.0	752	114.4
		1:32	20.6	21.2	678.4	102.9
	Low concentration	1:4	24.4	24.4	97.6	-
		1:8	12.2	13.6	108.8	111.4
		1:16	6.11	6.56	104.9	107.4
		1:32	3.06	3.91	125.1	127.7

Recovery values ranged from 94.1% to 99.4% for CgA and from 95.8% to 109.1% for S100A12, as shown in Table 3.

**Table 3:** Recovery of CgA and S100A12 in porcine hair extracts

	Sample (ng/mL)	Spiked analyte amount (ng/mL)	Expected* (ng/mL)	Detected (ng/mL)	Recovery (%)
CgA	76.6	500	288.3	286.6	99.4
	76.6	250	163.3	153.7	94.1
	76.6	125	100.8	95.2	94.4
	76.6	62.5	69.5	67.7	97.3
	76.6	31.3	53.9	53.1	98.5
S100A12	78.9	31.7	55.3	59.1	106.8
	78.9	15.9	47.4	50.9	107.4
	78.9	7.93	43.4	47.7	109.1
	78.9	3.97	41.4	40.2	97.1
	78.9	1.98	40.4	38.8	95.8

* Expected values were calculated as the mean of the sample and standard concentrations, since they were mixed at a 1:1 (v/v) ratio

Assays’ LOD and LLQ are represented in Table 4.

**Table 4:** Limit of detection (LOD) and low limit of quantification (LLOQ) for S100A12 and CgA assays

Analyte	LOD	LLOQ
S100A12 (ng/mL)	0.80	2.56
CgA (ng/mL)	15.34	22.83

### Effect of sex in S100A12, CgA, cortisol, and DHEA-S concentrations

The mixed-effects model revealed no significant main effect of sex on hair concentrations of CgA (F_1,18_ = 0.001, *p* = 0.98), S100A12 (F_1,54_ = 0.34, *p* = 0.56), cortisol (F_1,18_ = 0.01, *p* = 0.92), and DHEA-S (F_1,18_= 1.40, *p* = 0.25). In addition, no significant interaction between sex and production stage was observed for CgA (F_2,36_ = 1.83, *p* = 0.18), S100A12 (F_2,54_ = 1.72, *p* = 0.19), cortisol (F_2,36_ = 0.14, *p* = 0.87) or DHEA-S (F_2,36_ = 0.64, *p* = 0.53).

Consequently, data from both sexes were combined for subsequent analyses.

### Concentration of S100A12, CgA, cortisol, and DHEA-S in extracted hair across production stages

The significant differences observed in the concentrations of the four analytes across production stages for the entire cohort are presented in Fig. 1. In addition, Table 5 summarizes the median values, the 95% confidence intervals and the 25-75th percentiles for each production stage.

**Table 5:** Descriptive statistics and distribution parameters (Median, 95% CI, and 25-75th percentiles) for hair biomarkers across swine production stages

Biomarker	Stage	*n*	Median [95% CI]	25-75th percentiles
CgA (pg/mg)	L	20	433.5 [374.3–480.8]	371.1–511.1
	N	20	558.2 [474.2–714.9]	473.4–722.8
	F	20	636.2 [472.0–685.3]	467.6–768.0
S100A12 (pg/mg)	L	20	11.06 [7.48–34.91]	2.5–42.56
	N	20	114.1 [26.82–239.3]	25.30–260.1
	F	20	0.48 [0.00–14.95]	0.00–29.17
Cortisol (pg/mg)	L	20	53.34 [24.26–86.67]	27.36–83 − 34
	N	20	16.55 [13.33–20.67]	13.70–20.38
	F	20	18.52 [11.77–24.64]	11.84–24.30
DHEA-S (pg/mg)	L	20	76.92 [76.92–101.0]	76.92–122-6
	N	20	76.92 [76.92–76.92]	76.92–76.92
	F	20	320.5 [165.4–543.6]	159.8–545-5
Ratio Cortisol/DHEA	L	20	0.52 [0.31–0.95]	0.31–0.89
	N	20	0.21 [0.14–0.27]	0.15–0.27
	F	20	0.05 [0.02–0.15]	0.02– 0.13

Values are expressed as Median [95% Confidence Interval] and 25–75th percentiles.

n: number of subjects.

L: Lactation; N: Nursery; F: Fattening.

Hair S100A12 concentrations peaked at the end of nursery (N), showing a significant increase compared to the end of fattening (F) (Mean difference = 177.9 pg/mg; *p* = 0.0004) (Fig. 1A).

CgA concentrations showed an upward trend across the production cycle (Fig. 1B). Piglets at the end of lactation (L) showed the lowest CgA levels, which increased significantly at N (Mean difference = 85.5 pg/mg; *p* = 0.034) and remained significantly elevated at F (Mean difference = 177.7 pg/mg; *p* = 0.022).

Conversely, hair cortisol concentrations followed a descending pattern, presenting significantly higher values during lactation compared to both the subsequent nursery (Mean difference vs. L = 33.4 pg/mg; *p* = 0.0023) and fattening periods (Mean difference vs. L = 36.7 pg/mg; *p* = 0.0001,) (Fig. 1C).

DHEA-S levels remained low during L (Mean difference vs. F = 262.8 pg/mg; *p* = 0.0061) and N (Mean difference vs. F = 312.2 pg/mg; *p* = 0.0002) stages but experienced a significant increase at the end of fattening phase (Fig. 1D).

As a result of these distinct hormonal variations, the Cortisol/DHEA-S ratio progressively declined, showing significantly higher values at the end of lactation (Mean difference vs. F = 0.475 pg/mg; *p* = 0.0002) and nursery (Mean difference vs. F = 0.183 pg/mg; *p* = 0.02) compared to the fattening period (Fig. 1E).

### Correlations

The study revealed an absence of asignificant correlation between salivary and hair CgA concentrations when the same sampling times were matched (L, *r* = 0.24; N, *r* = 0.27; F, *r* = 0.22; *p* > 0.05).

No significant correlations were found between the different hair biomarkers (Spearman’s r ranging from − 0.24 to 0.14, all *p* > 0.05).

## Discussion

The present study provides novel knowledge about the use of pig hair as a biological matrix for assessment of neuroendocrine and immune-related biomarkers. Specifically, this work represents the first report about the immunoreactive detection of CgA and S100A12 in pig hair. It also confirms and extends previous findings on the measurement of cortisol and DHEA-S in this matrix. Together, these results contribute to highlight the potential of hair-based biomarkers for assessing animal welfare.

From an analytical perspective, measuring large protein biomarkers such as CgA and S100A12 in hair samples presents significant challenges due to the matrix specificity, compared to the traditional steroid hormones. While small, lipophilic steroids have been shown to readily incorporate into the hair shaft and are relatively easy to extract (Stalder and Kirschbaum 2012), the large molecular size and complex structure of proteins make their extraction from the highly keratinized hair matrix much more difficult, increasing the risk of matrix interference (Lee et al. 2006). In this line, further studies to determine the most appropriate method of hair extraction of CgA and S100A12 from hair should be performed. In hair extracts, the assays showed intra- and inter-assay imprecision below the recommended 20% (Food and Drug Administration, 2001), with CVs consistently < 15%. Alongside the high linearity (R^2^ = 0.99) and the adequate dilutional recovery (ranging from 94.1% to 109.1%), support the suitability of these assays for evaluating the immunoreactive detectability of CgA and S100A12 in hair extract samples obtained in our report. Regarding cortisol and DHEA-S measurement, although these steroids have previously been quantified in porcine hair (Bergamin et al. 2019; Scollo et al. 2025), to the authors’ knowledge, automated methods such as chemiluminescence technology, that have been used in this report, have not yet been used to measure them in this specific matrix. This automated technology could enhance analytical standardisation and reduce variability, advantages which are often lacking in manual assays.

At each sampling point, segments of hair of approximately 1 cm more closer to the skin of the same anatomical area were analysed. Based on porcine hair growth rates of 0.7 to 1.0 cm per month (Bacci et al. 2014; Prims et al. 2019), this segment would reflect the biomarker accumulation over the last 3 to 4 weeks prior to sampling. This standardization ensures that, in our report, measured concentrations are directly comparable across time points, representing the animals’ status during the final month of each stage.

Concentrations of CgA in pig hair showed a progressive increase from the end of lactation to the end of nursery and fattening. While these results should be treated with caution due to the methodological limitations of the assay of this report and the fact that they were obtained in only one trial on one farm; the higher CgA concentrations detected during the nursery phase may indicate a response to the challenges related with fattening . ). Although these stressors may be individually transient, their cumulative and recurrent nature over several weeks could potentially contribute to sustained sympathetic activation, thereby promoting greater incorporation of CgA into growing hair. This interpretation is consistent with previous studies reporting increased CgA concentrations in pigs exposed to repeated or prolonged environmental changes (Escribano et al. 2019; Huang et al. 2017). This finding provides additional support for the sensitivity of CgA in detecting physiological variation (Kanitz et al. 2019). The maintenance of elevated CgA concentrations during the fattening stage suggests that sympathetic activity may remain altered. Rather than a direct causal indicator of chronic stress, this could reflect a longitudinal adaptation to a range of management-related challenges, including social competition, higher stocking density and routine handling procedures, which have been widely described as potential stressors in commercial production systems (Oke et al. 2025). However, it should be pointed out that age-related factors or cumulative exposure throughout the production cycle may also influence these observed levels.

By contrast, S100A12 concentrations peaked at the end of nursery, followed by a significant decrease at the end of fattening. S100A12 is closely associated with innate immune activation and inflammatory processes, being released by activated neutrophils and monocytes (Xia et al. 2024). The elevated concentrations observed during the nursery period may reflect the heightened basal activation of the innate immune system that characterises this phase, even in clinically healthy piglets (Groot et al. 2021). The transition from milk to solid feed, together with age, changes in microbial exposure, and social environment, has been shown to induce transient alterations in immune and inflammatory pathways during the weeks following weaning (Campbell et al. 2013; Van Kerschaven et al. 2023). Previous studies have shown increased S100A12 concentrations in pigs subjected to acute stressors, such as transport to slaughter (Botía et al. 2023) and infections (Ortín-Bustillo et al. 2023), supporting its sensitivity to inflammatory and stress-related stimuli. The subsequent reduction in S100A12 concentrations during fattening may therefore be consistent with a progressive stabilisation of immune activity as the animals age and adapt to the finishing environment. However, while this dynamic could reflect innate immune activation following weaning, this remains speculative, as the present study does not include specific data on gut health, clinical status or inflammatory markers.

In this study, hair cortisol concentrations were significantly higher at the end of lactation than atthe end of nursery and fattening. This increase aligns with a previous report (Heimbürge et al. 2020), which found that two-week-old piglets had higher hair cortisol concentrations than ten-week-old or older piglets. It may also reflect the high physiological and metabolic demands of the lactation period, during which piglets experience rapid growth, nutritional dependence, and limited metabolic reserves (Noblet et al. 1997). Cortisol plays a central role in energy homeostasis by promoting gluconeogenesis, lipolysis, and glucose availability, thereby facilitating physiological adaptation to periods of high energetic demand (Mormède et al. 2007). In contrast, DHEA-S concentrations increased significantly at the end of fattening compared with lactation and nursery. DHEA is synthesised in the adrenal cortex and is closely associated with adrenal maturation and long-term steroidogenic capacity (Maninger et al. 2009). While species-specific differences must be considered and direct extrapolation from humans to pigs should be approached with caution, a rapid fall of DHEA concentrations after birth, followed by a gradual increase over time, has also been previously described in humans (Papadopoulou-Marketou et al. 2023).

Interestingly, our results demonstrated opposing trends between cortisol and DHEA-S concentrations. This inverse relationship can be explained by the biological role of DHEA-S, which plays well-known anti-glucocorticoid and neuroprotective functions and acts as an anabolic hormone that counterbalances the catabolic effects of cortisol during chronic stress (Lennartsson et al. 2012; McNelis et al. 2013). This dynamic balance is captured by the Cortisol/DHEA-S ratio, which was significantly higher during lactation. A high ratio may indicate a predominant catabolic state and higher physiological vulnerability, which further confirms the intense metabolic demands of early life (Erceg et al. 2025). Therefore, the higher DHEA levels observed during fattening, alongside decreasing cortisol levels, may reflect age-related endocrine maturation and a compensatory physiological mechanism designed to maintain homeostasis and promote growth, rather than only differences in stress exposure.

This study revealed no statistically significant correlation between salivary and hair CgA levels across production stages. This suggests that these matrices may reflect different information: hair reflects cumulative hormone exposure over extended periods, whereas saliva reflects transient hormonal fluctuations and may be influenced by circadian rhythms (Ortín-Bustillo et al. 2022a). This absence of correlation is consistent with the findings of previous studies in pigs that established that hair cortisol concentrations are not associated with single salivary or serum samples collected at corresponding time points (Prims et al. 2019). In addition, in our report, no correlations were found between the different hair biomarkers measured. This lack of correlation suggests that the long-term accumulation of these molecules may be driven by different physiological pathways, meaning that these analytes may provide complementary information. However, this hypothesis should be treated with caution. A lack of correlation does not prove that the biological roles are independent; it could also reflect differences in the biomarker kinetics, their specific incorporation rates into the hair shaft, variable matrix effects, or analytical variability among the different assays.

This report has several limitations that should be noted. Firstly, the extraction protocol involved a methanol-based approach to enable the simultaneous profiling of lipophilic steroids and targeted proteins from a single hair sample. However, methanol is an organic solvent that can alter the complex tertiary structure of intact glycoproteins (CgA) and calcium-binding proteins (S100A12). In addition, the procedure included a complete evaporation step followed by the reconstitution of the dry residue in PBS, which transitions the analytes into a physiologically compatible aqueous environment. Both methanol and the evaporation could produce changes in the proteins and, consequently, rather than measuring fully intact proteins, the assay could detect preserved immunoreactive fragments or partial antigenic structures (epitopes) that remain stable and are successfully recognized by the antibodies. Further studies comparing different extraction protocols are needed to determine whether there are better approaches to extract these proteins. Until such studies have been conducted, the practical application of these analytes in hair will be limited. Secondly, recovery experiments should ideally have been performed to determine the extraction efficiency of the method in pig hair. However, validating this procedure is difficult because it is hard to accurately determine the extraction yield, since adding an external substance to the hair does not mirror its natural incorporation into the hair structure. This is the case for cortisol, the analyte most frequently measured in hair, where it is recognised that accurately representing endogenous hair cortisol by spiking a hair sample is unfeasible (Slominski et al. 2015). Due to this limitation, our recovery experiments were performed post-extraction by adding exogenous materials (CgA-BSA and serum S100A12) to the liquid extracts. As these materials may not perfectly mimic native proteins deeply embedded within the hair, our recovery values reflect the absence of assay interference in the final liquid extract rather than the true extraction efficiency from the solid matrix. Specifically, the use of serum-derived or protein-conjugated standards may not be enough to account for the potential degradation or structural alterations that occur when proteins are incorporated into hair over time or during the extraction process. Consequently, without a definitive evaluation of true extraction efficiency, the reported absolute concentrations should be interpreted with caution and be considered relative estimates reflecting immunoreactive detectability. Furthermore, as these are novel biomarkers in this matrix, future studies should evaluate analyte stability during storage and processing, and determine whether these large proteins originate strictly from the systemic circulation or also from local sources, such as the sweat or sebaceous glands. Additionally, this study focused on a relatively small cohort (*n* = 20) of pigs from a single farm, evaluating changes only during a specific production cycle. Therefore, these results should be approached cautiously, as environmental, management, and health-related variability across different production systems could significantly influence biomarker profiles. Further studies involving a larger number of individuals from various farms in different production stages are needed to determine the practical value of measuring these analytes for detecting stress or welfare problems in general.

## Conclusion

 This report showed that there is immunoreactive detectability of CgA and S100A12 in the hair extracts of pigs. Hair sampling causes minimal disturbance to the animals, which is a clear advantage over conventional sampling methods. However, this report should be considered a preliminary proof-of-concept study due to the relatively small sample size, the single-farm design, and current uncertainties regarding extraction efficiency and protein incorporation mechanisms. These factors may restrict the general applicability of the results. Further studies should clarify these aspects and evaluate whether assessing these analytes could provide complementary information on sympathetic activation and immune status, offering a more integrative view of the physiological changes associated with pig production stages.

## Data Availability

No datasets were generated or analysed during the current study.
